# Comparison of the effectiveness of ultrasound-guided versus fluoroscopy-guided medial lumbar bundle branch block on pain related to lumbar facet joints: a multicenter randomized controlled non-inferiority study

**DOI:** 10.1186/s12871-023-02029-9

**Published:** 2023-03-11

**Authors:** Marie-Laure Nisolle, Djamal Ghoundiwal, Edgard Engelman, Walid El Founas, Jonathan Gouwy, Emmanuel Guntz, Panayota Kapessidou, Turgay Tuna

**Affiliations:** 1 Department of Anesthesiology and Pain Medicine, Erasme Hospital, Université Libre de Bruxelles (ULB), Route de Lennik 808, 1070 Brussels, Belgium; 2 Department of Anesthesiology and Pain Medicine, CHU Saint-Pierre, Université Libre de Bruxelles (ULB), Rue aux Laines 105, 1000 Brussels, Belgium; 3 EW Data analysis, Brussels, Belgium; 4 Department of Anesthesiology and Pain Medicine, Braine l’Alleud Hospital, Université Libre de Bruxelles (ULB), Rue Wayez 35, 1420 Braine-l’Alleud, Belgium

**Keywords:** Low-back pain, Chronic pain, Ultrasonography, Fluoroscopy, Nerve block, Zygapophyseal joint, Lumbar facet joint syndrome

## Abstract

**Background:**

The aim of this multicenter randomized interventional prospective study was to compare the ultrasound (US)-guided lumbar medial branch block (LMBB) with the fluoroscopy (FS)-guided LMBB in terms of analgesic efficacy and disability in the setting of the treatment of pain arising from the lumbar facet joints (LFJ).

**Methods:**

Fifty adults with a “LFJ” syndrome were randomized into two groups: in group FS, fluoroscopic-guidance was used to block the medial branch at three lumbar levels (L3-L4, L4-L5 and L5-S1); in group US, same blocks were performed under ultrasound. Needle transverse approach was used with both techniques. Effects of these procedures were assessed with a Visual Analogue Pain Scale (VAPS), the Oswestry Disability Index (ODI) and the Duke’s Activity Status Index (DASI) scale, before the treatment, 1 week and 1 month after. Hospital Anxiety and Depression Scale (HADS) score was also collected before the procedure. Analysis of variance, one (for non-inferiority) and two-sided Mann-Whitney tests and Chi-square tests were performed.

**Results:**

LMBB under US-guidance was not inferior to FS-guidance (*P* = 0.047) in terms of VAPS, ODI and DASI at 1 week and 1 month*.* Duration of techniques and HADS were similar between groups (=0.34; *p* = 0.59).

**Conclusions:**

The medial lumbar bundle branch block under ultrasound-guidance is not inferior to the fluoroscopy-guidance procedure in effectively alleviating pain arising from the facet joints. Considering that this ultrasound technique has the benefit of an irradiation-free, real-time procedure, it can be considered as an effective alternative to the fluoroscopy-guided technique.

## Introduction

Lumbar facet joints (LFJ) syndrome is a common source of spinal suffering affecting up to 45% of patients with a chronic low back pain (LBP) because of inflammation, degenerative or arthritic changes, overload of the posterior LFJ or muscle imbalance [1–6]. It can be defined as a lumbosacral pain sometimes associated with sciatic pain that spread from any structure of facet/zygapophyseal joints [1–6]. Despite the high prevalence and the annual cost that it can generates, this syndrome is always difficult for pain doctors to take care of [5].

In fact, it cannot be identified by imaging or physical examination alone, because of various presentations (often pain exacerbated by lumbar extension, lateral flexion or rotation) and referred pain, making the diagnosis complicated [1, 7, 8].

The first-line therapy for the alleviation of chronic LBP of zygapophyseal joint origin includes bedrest, oral analgesics/anti-inflammatory drugs and physical therapy. If unsuccessful, intra-articular injections and lumbar medial branch block (LMBB) can provide diagnosis and are always performed prior to the neurotomy (radiofrequency or cryoneurolysis) which is able to provide short and long-term pain relief [1, 2, 4–6, 9–13]. The LMBB targets the medial branches of the posterior rami. It blocks sensory nerve branches of the inter-facet joint (coming from the two levels involved with an exception for L5 for which an ascending articular branch originates only to the L5-S1 joint), periosteum of the neural arch, nociceptors, ligaments and muscles (interspinous and multifidus) [1, 2, 14, 15].

According to pain centers, LMBB is realized by fluoroscopy (FS), ultrasonography or computed tomography (CT) scanner for guidance [7]. The current standard technique is fluoroscopy (transverse-axial plane), but the ultrasound (US) technique is also described in this plane [13]. Both procedures require three puncture points. Some studies have reported similar outcomes with both equipments but US seems the most interesting method due to the fact that this technique is free from irradiation, mobile, provides real-time imaging and is cost-effective [3, 5–8, 14, 16]. Further, US-guided LMBBs have shown a high success rates, less complications and has been validated in cadaver studies [6]. The needle placement and clinical efficiency under US-guidance is also compared with CT and validated in a study by Ye et al. [7]. Nowadays, only one retrospective study by Han et al. has compared the efficacy of LMBB performed under FS vs US reporting similar results for both techniques [6].

The aim of the present study was to prospectively compare the effectiveness of those two imaging-guided techniques regarding the early and long term pain scores.

## Materials and methods

### Design and population

This prospective multicenter randomized controlled trial was conducted from 6 January 2021 until 10 March 2022 in three pain management centers in Brussels (Belgium); Saint-Pierre University Hospital (César de Paepe site), Erasme and Braine-l’Alleud Hospitals. This open label study was approved by the three Ethics Committees of ULB (main center is Erasme Hospital; P2020/557, 31th of December 2020 - B4062020000221) and was registered on the publicly accessible Clinical Trial Registry (NCT04658953, 09/12/2020).

All participants received and signed an informed consent before the infiltration including all the information about the procedure, the benefits and the possible risks (hematoma, infection, vasovagal syncope, intrathecal injection and spinal anesthesia). Allocation concealment was achieved using opaque numbered sealed envelopes, opened only after each patient’s arrival in the operating theatre, in each center. Patients beneficiated the FS or the US technique, according to the randomization and both the attending anesthetist and the patient were aware of the allocated technique. All patients enrolled in the study were requested to report to investigators the evolution of the applied scores variables by phone at the predetermined time points.

Inclusion criteria: patients aged between 20 and 80 years old, having a chronic LBP compatible with a LFJ syndrome for at least 3 months without response to a conservative treatment of minimum 4 weeks.

Exclusion criteria: refusal to participate to the study, pregnant or breastfeeding women, allergy to injected products (Methylprednisolone (Depomedrol) or Lidocaine (Linisol)), psychiatric disorders hindering understanding of the protocol, local or systemic infection or coagulation disorder. In addition, patients should not present any sign of dissociated pain, radiculitis, neurological diseases (including stroke, Parkinson’s disease), spinal instability or deformities (such as scoliosis, ankylosing spondylitis), history of lumbar surgery, fracture or lumbar tumor. Further, patients with a body mass index (BMI) > 35 kg/m^2^ have not been included because of a predisposition to early degeneration of articular structures and the risk of bad visualization by US, the resolution decreasing with the depth of the fat layers.

For this study, the investigators have blocked the LMBB on three lumbar levels (L3-L4, L4-L5 and L5-S1) performed under US versus FS according to a transverse approach. The aim was to compare the equivalence of these two modalities by the evaluation of the benefit of the procedures on pain with a Visual Analogue Pain Scale (VAPS), the evolution of the possibility of carrying out daily activities with the Duke’s Activity Status Index (DASI) and the limits of daily activities with the Oswestry Disability Index (ODI). Scores were collected before injection, 1 week and 1 month after. Hospital Anxiety and Depression Scale (HADS) before the procedure was also filled to detect anxiety and depressive disorders in the setting of the treatment of pain arising from the lumbar facet joints. Moreover, duration of the procedures, incidence of adverse events, work status and medications were obtained**.**

### US-guided and FS-guided infiltrations

Patients were positioned in prone position with a pillow under the stomach to facilitate the view of the structures by compensating the lumbar lordosis. After disinfection of the lumbo-sacral region with alcoholic chlorhexidine 0,5%, an infiltration was performed in three puncture points unilaterally (L3-L4, L4-L5 and L5-S1 levels) with a 22-gauge spinal needle in the transverse plane. At each level, 1 mL of a mixture consisting of 3 mL of Linisol 2% (60 mg of lidocaine) plus 1 mL of Depomedrol 40 mg/Lidocaine 10 mg was injected. The procedure time was recorded from insertion to removal of the needle. The ultrasound devices used were different between the centers; C6–2 probe of the Sparq ultrasound system (Philips N.V., Netherlands) at César de Paepe Hospital, C1–5 probe of the Logiq S8 ultrasound system (GE Healthcare Inc., Illinois, United States) at Erasme and Braine-l’Alleud Hospital. The scopies used were also different; Ziehm brand (Ziehm Inc., Florida, United States) at César de Paepe and Siemens brand (Siemens Healthcare Gmbh, Germany) at Erasme and Braine-l’Alleud Hospitals. It should be noted that thermocoagulation was performed after 2 weeks on patients at the Braine-l’Alleud hospital.

**Fig. 1:**
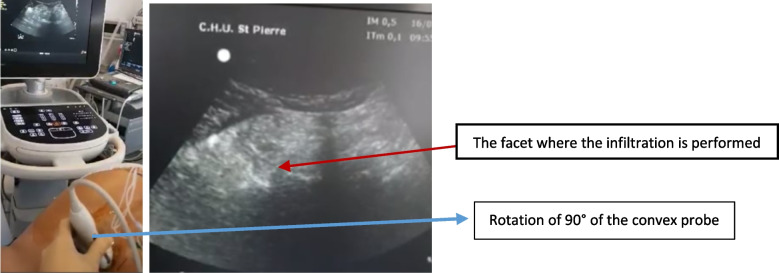
Firstly, the probe was placed longitudinally in order to have the facet paramedian sagittal view (wave image). Secondly, a rotation of 90° was made to be in the transverse plane

In the US group, once the spinous processes of the lower lumbar vertebrae were located, with a convex probe with the mark on the cranial side (Fig. 1), the probe was tilted of 90° and the needle was introduced trice in a transverse axis, towards the midline in the direction of the median bone contacts of L3, L4 and L5.

In the FS group, The C-shaped arm of a X-ray fluoroscopy was positioned around the patient in an antero-posterior view tilted ¾ in order to see the classic view called “scotty dog”. The puncture point was determined by the positioning of the needle with the so-called “tunnel vision”. The needle was thus brought to the bone contact corresponding to the eye of the scotty dog in tunnel vision, corresponding to the area of passage of the lumbar median branch.

### Statistical analysis

The primary endpoint was to investigate the non-inferiority of the US group, comparing to the FS group in regard to the evolution of the ODI measured 1 month after the LMBB. Power analysis was performed with a projected standard deviation of 7. If there was truly no difference between the standard and experimental treatment, then 50 patients are required to be 80% sure that the lower limit of a one-sided 95% confidence interval (or equivalently a 90% two-sided confidence interval) will be under the non-inferiority limit of + 5 points. Analysis of non-inferiority was realized with a one-sided Mann-Whitney test.

All data for the ODI, the VAPS and the DASI measured before, 1 week and 1month after the LMBB were compared by an analysis of variance for repeated measures with mixed models with treatment arms, time, thermocoagulation status, time x treatment interaction, thermocoagulation status x time interaction and time x treatment x thermocoagulation status interaction terms.

Categorical data were compared by a χ^2^ test and the other continuous non-longitudinal variables such as HADS were compared with the Mann–Whitney test. Data were presented as mean ± SD or number (%). For all tests, a *P* value less than 0.05 was considered statistically significant. All analyses were performed using the NCSS 21.0.4 statistical package (NCSS, LLC; Kaysville, Utah, USA).

## Results

**Fig. 2:**
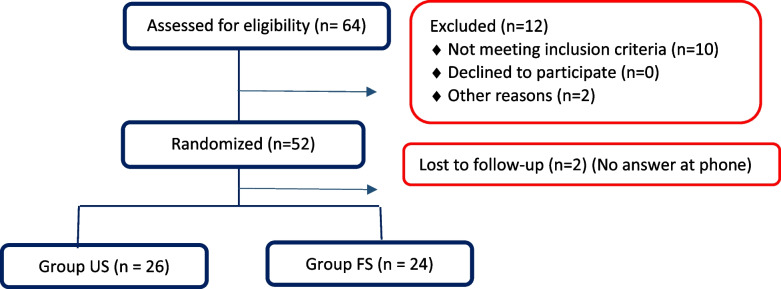
Illustration of the participant flow

Investigators screened 64 patients during the study period. Of them, 50 patients who answered to the phone call participated until the end of the study and were considered for the per protocol analysis (Fig. 2).

Patients’ characteristics were shown in Table 1. 46% of patients have pain bilaterally and duration of techniques shows no statistical difference between the groups (*p* = 0.34). Both techniques improved VAPS, ODI and DASI at any time without statistical difference between both groups (*p* > 0.05) (Table 2 and Table 3). There was no difference for HADS between both groups (Table 4) and no correlation between HADS and VAPS before the infiltration. The primary end-point was validated (*p* = 0.047) (Table 5). No side effects were reported during the study period.

**Table 1:** Patient characteristics

	US group (***n*** = 26)	FS group (***n*** = 24)	***P***-value Mann-Whitney or Chi-square test
Age (years)	57.9 ± 13.6	54.8 ± 14.9	0.40
Sex (male / female)	9 (35%) / 17 (65%)	6 (25%) / 18 (75%)	0.46
BMI (kg/m^2^)	27.8 ± 4.2	26.3 ± 3.7	0.27
Working (yes/no)	6 (23%) / 20 (77%)	6 (25%) / 18 (75%)	0.87
Unilateral / bilateral pain	13 (50%) / 13 (50%)	14 (58%) / 10 (42%)	0.55
Anti-inflammatory usage	7 (27%)	10 (42%)	0.27
Opioid usage	9 (35%)	11 (46%)	0.42
Procedure time (seconds)	101.8 ± 50.2	104.3 ± 31.0	0.34

Data are mean ± SD or number of patients (%)

**Table 2:** Number of patients improving at one week and at one month after LMBB

VAPS		ODI		DASI	
1 week	1 month	1 week	1 month	1 week	1 month
38 patients (74.5%)	38 patients (74.5%)	35 patients (68.6%)	40 patients (78.4%)	14 patients (27.4%)	18 patients (35.3%)

**Table 3:** Between and within groups comparisons of pre- and post-injection pain relief characteristics and functional assessment

		Before LMBB	1 week after LMBB	1 month after LMBB
**VAPS**	**US-group**	5.9 ± 2.0	4.1 ± 2.0**	3.7 ± 2.2**
	**FS-group**	6.9 ± 1.5	4.7 ± 2.6**	4.3 ± 2.9**
	**US-group + FS-group**	6.4 ± 1.8	4.4 ± 2.3***	4.0 ± 2.5***
US-group versus FS-group: *p* = 0.23 Time: *p* < 0.000001 Thermocoagulation YES x Thermocoagulation NO: *p* = 0.28 Group x Time interaction: *p* = 0.77 Thermocoagulation status x Time interaction: ***p*** **= 0.046** Group x Thermocoagulation status x Time interaction: *p* = 0.98				
**ODI** (points)	**US-group**	20.6 ± 8.2	15.8 ± 8.5**	15.2 ± 8.4**
	**FS-group**	20.1 ± 8.5	17.3 ± 9.3	15.4 ± 10.2**
	**US-group + FS-group**	20.3 ± 8.3	16.5 ± 8.8***	15.1 ± 9.2***
US-group versus FS-group: *p* = 0.85 Time: *p* < 0.000001 Thermocoagulation YES x Thermocoagulation NO: *p* = 0.12 Group x Time interaction: *p* = 0.65 Thermocoagulation status x Time interaction: *p* = 0.54 Group x Thermocoagulation status x Time interaction: *p* = 0.69				
**DASI** (points)	**US-group**	24.3 ± 12.1	27.6 ± 12.6	27.0 ± 11.0
	**FS-group**	27.7 ± 11.1	28.1 ± 11.4	31.2 ± 12.7
	**US-group + FS-group**	25.9 ± 11.6	27.8 ± 11.9	29.0 ± 11.9**
US-group versus FS-group: *p* = 0.24 Time: *p* = 0.005 Thermocoagulation YES x Thermocoagulation NO: *p* = 0.92 Group x Time interaction: *p* = 0.13 Thermocoagulation status x Time interaction: *p* = 0.62 Group x Thermocoagulation status x Time interaction: *p* = 0.10				

Analysis of variance for repeated measures by Mixed Models procedure

Bonferroni probability level: Inside each group

* p < 0.05 vs *Before block*

** p < 0.001 vs *Before block*

*** p < 0.00001 vs *Before block*

**Table 4:** Hospital Anxiety and Depression Scale (HADS)

	US group	FS group	*P*-value Mann-Whitney test
HADS anxiety	9.8 ± 5.2	10.6 ± 4.7	0.61
HADS depression	6.7 ± 4.6	6.7 ± 4.7	0.94
HADS total	16.3 ± 9.3	17.3 ± 8.8	0.59

Data are mean ± SD

**Table 5:** Non-inferiority of ODI at 1 month after LMBB

US-group	FS-group	Difference between the groups [95% CI for the difference]	Mann-Whitney U test for non-inferiority	Conclude non-inferiority at α = 0.05
15.1 ± 8.4	15.0 ± 10.1	0.108 [−5.2; + 5.4]	**0.047**	**Yes**

Data are mean ± SD

Non-inferiority margin for mean value of US-guided group: 5 points above FS-guided group

Concerning the thermocoagulation performed after 2 weeks at the Braine-l’Alleud Hospital, the analysis of variance for repeated measures with mixed models showed a statistical significant interaction between thermocoagulation status and time (*p* = 0.046), with a smaller, although not significant (*p* = 0.08- Bonferroni test) mean value for VAPS after 1 month in patients with thermocoagulation. This led us to suspect a possible effect of thermocoagulation on VAPS after 1 month. However, the treatment group x thermocoagulation x TIME interaction was not significant (*p* = 0.69) showing that the evolution of the VAPS values over time in both treatment groups was identical whatever the thermocoagulation status (Table 6).

**Table 6:** Visual Analogue Pain Scale (VAPS)

	No thermocoagulation *N* = 34	Thermocoagulation *N* = 16	*P*-value Bonferroni test
Before	6.4 ± 1.8	6.4 ± 2.0	1.0
1 week after LMBB	4.5 ± 2.3	4.2 ± 2.3	1.0
1 month after LMBB	4.5 ± 2.5	2.9 ± 2.4	0.08
	Analysis of variance for repeated measures by Mixed Models: Thermocoagulation x Time interaction: ***p*** **= 0.046**		

Data are mean ± SD

## Discussion

This prospective randomized study showed that the LMBB under US-guidance is not inferior to FS-guidance. It confirmed the retrospective results of Han et al. [6], suggesting that in the context of the conservative management of articular pain of the lower lumbar facets, ultrasound is to be considered. In addition, these results are also confirmed by the retrospective study by Ha et al [5]. Both techniques improve VAPS, ODI and DASI equally at 1 week and at 1 month. Most patients were very satisfied with the LMBB which has a real benefit on LBP, for functional abilities and the performance of daily activities (personal care, handling of loads, walking, sitting or standing, sleep, social life, travel etc). There is no correlation between anxiety and depression (HADS) and pain assessment (VAPS). Similarly to Meucci et al. [17], in our work, more women are affected by the LFJ syndrome which could be due to the physical (less muscle, often more workload) and psychological characteristics. There are no complication reported during the study but based on the review by Cohen et al., these are extremely rare (10).

Few patients are no longer taking medications and have psychological follow-up. The pain is not always treated optimally in the “bio-psycho-social” model required for chronic pain. Chronic LBP is often multifactorial and is a major health care problem. Accordingly, more attention should be paid to it in order to avoid depression, anxiety and work stoppage (76% of our patients no longer work), which has a major socio-economic impact and sometimes leads to a sedentary lifestyle causing muscle weakness, eliciting a vicious circle of risk of chronic pain [2, 10, 12, 17]. Interestingly, some specific exercises, yoga, osteopathy and acupuncture have significantly reduced LBP. This should be considered, in addition to a multidisciplinary approach centered on the bio-psycho-social model [10].

**Fig. 3:**
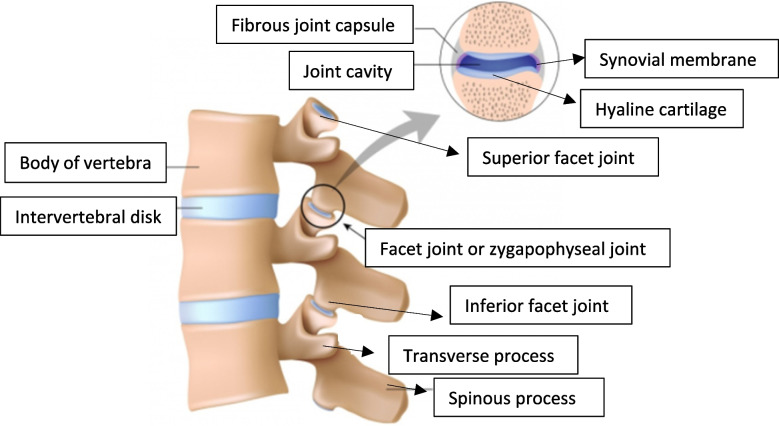
Facet joints of spine’s anatomy. Facet syndrome is a pain that can originate from any structure of the facet joints; fibrous capsule, synovial membrane, hyaline cartilage and bone

As a reminder, five vertebrae constituting the lumbar spine are separated anteriorly by the discs which can be a source of pain and posteriorly by the LFJ also called zygapophyseal joints, composed of the superior articular process and inferior articular process. Between these articular processes, lies the synovial membrane, a hyaline cartilage covered by the fibrous capsule within the lumbar spine (Fig. 3) [4, 14]. The LMBB is an infiltration of the medial branch of the posterior rami. This nerve is responsible for the transmission of the pain generated by the zygapophyseal joint; the block is performed in order to confirm the origin of the pain. In 10–15% of cases, it can provide lasting relief. The effects often appear after a few days (typical of corticosteroids) and if there is at least 50% improvement during a limited period, denervation can be considered with a high frequency electric current. This technique is called thermocoagulation.

LMBB is a diagnostic block but can sometimes be considered as therapeutic due to 3.5 months average pain relief of LBP and the significant improvement in functional capacity [2, 9]. If there is an improvement in pain of at least 50% after the first infiltration of LMBB, patient may benefit from thermocoagulation of LFJ nerves within 3 months. It allows prolonged pain relief and it reduces disability and the need for painkillers [12, 17].

Local anesthetics were injected since it blocks the sympathetic reflex arc and the axonal transport inside nerve fibers. Furthermore, it suppresses nociceptive discharge with anti-inflammatory effect. In addition, we use corticosteroids for their anti-inflammatory, immunosuppressive and anti-edematous effects with an inhibitory action of neuronal transmission in the C fibers [2, 12].

Even if US-guidance does not clearly detect a foraminal extent and an intravascular injection, these complications have also been described with the technique by FS-guidance [16]. Recently, US have even shown fewer complications [6]. Moreover, it is a reliable alternative for LMBB because it is cost-effective, available, widely used and portable, allowing to follow the progress of the needle in real-time without ionizing radiation. This latter aspect is particularly interesting with pregnant patients. Although Greher et al. [16] mention that images are appropriate with a BMI of 36 kg/m^2^, it is preferable to use fluoroscopy when there is poor visualization due to deeper layers.

As limits to our study, we note a small population sample with an age variability implying anatomical distinctions, a relatively short follow-up of 1 month and the different ultrasound and fluoroscopy machines between the centers. More, patients often have other complaints than pain unilateral LFJ syndrome (such as bilateral pain, pain in knees, hips, rheumatoid arthritis, fibromyalgia etc): this lack of homogeneity is certainly unavoidable when considering the types of patients who visit pain clinics. Also, patients report changes in VAPS levels over the course of the day and according to their activities so all of that biases the rating scales. Although thermocoagulation is performed after 2 weeks at Braine-l’Alleud Hospital, it has no influence on the comparison between the two techniques.

Despite the benefit of LMBB, there are few articles comparing imaging methods for this infiltration as well as on improving the quality of life of patients after the procedure, which is essential. For future investigations, we propose the comparison of LMBB performed by FS versus US with the US-guidance in a longitudinal axis (as proposed by Chang et al. but not yet validated) which requires only one puncture instead of three, which would be an advantage for patient (faster and less painful) [14]. Further, this method would be interesting for L4-L5 and L5-S1 level due to the limited visualization of the needle at the L5 level by the iliac crest in the transverse view [13].

## Conclusion

The present study shows that the medial lumbar bundle branch block conducted using ultrasound-guidance is not inferior to the fluoroscopy-guidance procedure in terms of pain relief and functional improvement. Considering that ultrasound has similar benefits, is a non-irradiating technique, easier to handle and allows real-time manipulations, it should be considered as an adequate and safe alternative to the fluoroscopy-guided technique.

## Data Availability

The datasets used and analysed during the current study available from the corresponding author on reasonable request. The data will be anonymized.
