# Correction: Comparison of the effectiveness of ultrasound-guided versus fluoroscopyguided medial lumbar bundle branch block on pain related to lumbar facet joints: a multicenter randomized controlled noninferiority study

**DOI:** 10.1186/s12871-023-02110-3

**Published:** 2023-05-08

**Authors:** Marie‑Laure Nisolle, Djamal Ghoundiwal, Edgard Engelman, Walid El Founas, Jonathan Gouwy, Emmanuel Guntz, Panayota Kapessidou, Turgay Tuna

**Affiliations:** 1 Department of Anesthesiology and Pain Medicine, Erasme Hospital, Universite Libre de Bruxelles (ULB), Route de Lennik 808, Brussels, 1070 Belgium; 2 Department of Anesthesiology and Pain Medicine, CHU Saint-Pierre, Université Libre de Bruxelles (ULB), Rue aux Laines 105, Brussels, 1000 Belgium; 3 EW Data analysis, Brussels, Belgium; 4 Department of Anesthesiology and Pain Medicine, Braine l’Alleud Hospital, Université Libre de Bruxelles (ULB), Rue Wayez 35, Braine-l’Alleud, 1420 Belgium

**Correction: BMC Anesthesiol 23, 76 (2023)**

**https://doi.org/10.1186/s12871-023-02029-9**

Following publication of the original article [1], the authors reported an error in affiliations 2 and 4. The term ULB has been expanded to “Université Libre de Bruxelles”.

The affiliations has been updated above and the original article [1] has been corrected.
